# Baseline NT-proBNP levels as a predictor of short-and long-term prognosis in COVID-19 patients: a prospective observational study

**DOI:** 10.1186/s12879-024-08980-3

**Published:** 2024-01-08

**Authors:** Diana Mojón-Álvarez, Teresa Giralt, José Carreras-Mora, Alicia Calvo-Fernández, Andrea Izquierdo, Cristina Soler, Paula Cabero, Silvia Pérez-Fernández, Beatriz Vaquerizo, Núria Ribas Barquet

**Affiliations:** 1 Cardiology Department, Hospital del Mar, Passeig Marítim de la Barceloneta 25-29, Barcelona, 08003 Spain; 2 Medicine Department, Autonomous University of Barcelona, Bellaterra, Barcelona, 08193 Spain; 3 Medicine Department, Pompeu Fabra University, Barcelona, 08005 Spain; 4 Scientific Coordination Facility, Biocruces Bizkaia Health Research Institute, Barakaldo, 48903 Spain; 5 Heart Disease Biomedical Research Group (GREC), IMIM (Hospital del Mar Medical Research Institute), Barcelona, 08003 Spain; 6 CIBER of Cardiovascular Diseases (CIBERCV), Instituto de Salud Carlos III (ISCIII), Madrid, 28029 Spain

**Keywords:** COVID-19, SARS-CoV-2, Prognosis, Biomarkers, NT-proBNP

## Abstract

**Background:**

Up to 20% of COVID-19 patients can suffer COVID-19-related myocardial injury. Elevated cardiac biomarkers, such as hs-cTnT and NT-proBNP, have been related to worse short-term prognosis. However, data on NT-proBNP and long-term prognosis are scarce. We have evaluated the potential association of baseline age-adjusted NT-proBNP levels and outcomes at one-year follow-up in COVID-19 patients.

**Methods:**

This was a single-center prospective study of 499 COVID-19 patients in whom NT-proBNP was assessed at hospital admission. NT-proBNP levels were age-adjusted and patients were classified as high or low NT-proBNP. Clinical and demographic characteristics, comorbidities, laboratory results, and in-hospital complications and mortality were compared between the two groups. Survivors of the acute phase of COVID-19 were followed up for one year from admission to detect readmissions and mortality.

**Results:**

The 68 patients with high NT-proBNP levels at hospital admission were older, with more cardiovascular risk factors, cardiovascular disease, comorbidities, myocardial injury, and higher levels of inflammatory markers than patients with low NT-proBNP levels. They also had more in-hospital complications and a higher acute-phase mortality rate (60.3% vs. 10.2%, *p* < 0.001). High NT-proBNP levels were an independent marker of death during hospitalization (HR 1.95; CI 1.07–3.52). At one-year follow-up, high NT-proBNP levels were independently associated with mortality (HR 2.69; CI 1.47–4.89). Among survivors of the acute phase of COVID-19, there were no differences in hospital readmissions between those with high vs. low NT-proBNP levels, but survivors with high baseline NT-proBNP levels showed a higher 1-year mortality rate (7.4% vs. 1.3%, *p* = 0.018).

**Conclusions:**

High age-adjusted NT-proBNP levels at the time of hospital admission for COVID-19 are associated with poor short and long-term prognosis. High NT-proBNP seems also to be related to worse prognosis in survivors of the acute phase of COVID-19. A closer follow-up on these patients may be crucial.

**Supplementary Information:**

The online version contains supplementary material available at 10.1186/s12879-024-08980-3.

## Introduction

Severe acute respiratory syndrome coronavirus 2 (SARS-CoV-2), also known as coronavirus disease 2019 (COVID-19), was declared a global pandemic in March 2020, with significant mortality and morbidity worldwide [1, 2]. The main manifestations of COVID-19 range from mild to severe respiratory conditions, including pneumonia and acute respiratory distress syndrome (ARDS), but it can also affect other systems.

Mortality is particularly high in certain patient groups, such as older patients and those with cardiovascular risk factors [3, 4]. Since the beginning of the pandemic, different biomarkers have been analyzed in order to identify patients with a high risk of complications [5–7]. Up to 20% of COVID-19 patients experience myocardial injury, defined as a high-sensitivity cardiac troponin T (hs-cTnT) value over the 99th percentile URL (upper reference limit). Different mechanisms have been posited for this myocardial injury, including inflammation, hypoxemia, direct viral myocardial infection, plaque rupture leading to myocardial infarction, and myocardial stress that may result in heart failure [8, 9].

N-terminal pro B-type natriuretic peptide (NT-proBNP), a biomarker of hemodynamic myocardial stress and heart failure, plays a crucial role in the pathophysiology of heart failure and is widely accepted as a marker cardiac disease severity [10]. NT-proBNP is also frequently elevated among patients with sepsis and severe inflammatory or respiratory illnesses [11–13], and it has been associated with unfavorable outcomes in ARDS patients [11].

Among COVID-19 patients, hs-cTnT has been related to worse short-term [9] and long-term prognosis [14–16]. NT-proBNP has been associated with poorer short-term prognosis [17–20], but data are scarce regarding the potential association between baseline NT-proBNP values and long-term prognosis. In order to shed light on the impact of baseline NT-proNP values in COVID-19 patients, we have examined the association of baseline NT-proBNP with in-hospital complications and mortality, as well as with hospital readmission and mortality in survivors of the acute phase of COVID-19.

## Methods

### Study design and data collection

This was a prospective, observational, single-center study of COVID-19 patients diagnosed and treated at Hospital del Mar, Barcelona, Spain, from February 27 to April 7, 2020. The only exclusion criterion was the absence of baseline NT-proBNP values.

COVID-19 infection was confirmed by reverse-transcription polymerase chain reaction. Clinical follow-up was performed during the hospital stay and for one year after hospital admission. The acute phase of COVID-19 was defined as the time between the initial hospital admission and hospital discharge. Death due to any cause – either in hospital or after discharge – and hospital readmission after discharge were recorded. The following clinical and demographic characteristics were entered in an electronic database: age, sex, smoking history, comorbidities, cardiovascular risk factors, body mass index (BMI), chronic kidney disease (CKD), prior history of coronary disease, atrial fibrillation, chronic heart failure, chronic obstructive pulmonary disease (COPD), cerebrovascular disease, cancer, and peripheral vascular disease. Obesity was defined as BMI > 30 kg/m. CKD was defined as an estimated glomerular filtration rate < 60 ml/min/1.73m^2^.

The following parameters were also entered in the data base: cardiac biomarkers, hs-cTnT, NT-proBNP, C-reactive protein, lactate dehydrogenase (LDH), D-dimer, blood count, kidney function, chest radiography, electrocardiographic findings, treatments, and in-hospital complications. All patients who survived the acute phase of COVID-19 were followed up for one year after their initial hospital admission through telephone contacts, face-to-face visits, and electronic medical records. Hospital readmission was defined as a hospital stay longer than 24 h. Cardiac readmission was considered if it was due to coronary event, heart failure or arrhythmia. Mortality was classified as cardiovascular, non-cardiovascular or unknown.

The study complied with the Declaration or Helsinki and clinical practice guidelines. It was approved by the ethics committee and the research commission of our hospital (CEIm number 2020/9178). All patients gave their signed informed consent.

### NT-proBNP assessment

NT-proBNP levels were measured using an electrochemiluminescence immunoassay “ECLIA” system (Elecsys 2010, Roche Diagnostics Ltd., Mannheim, Germany). The blood test, including NT-proBNP values, was conducted within the first 24 h from admission.

In accordance with current clinical practice guidelines [21], NT-proBNP values were considered high if serum levels were above the age-adjusted cut-off point for ruling out acute heart failure: in patients < 50 years, the cut-off was ≥450 pg/ml; in patients 50–75 years, the cut-off was ≥900 pg/mL; in patients > 75 years, the cut-off was ≥1800 pg/mL [22, 23].

### Aims

The primary aim of our study was to assess mortality at the 1-year follow-up based on baseline age-adjusted NT-proBNP values. Secondary aims included evaluating in-hospital mortality, in-hospital complications, and the occurrence of hospital readmission or mortality after hospital discharge in patients who survived the acute phase of COVID-19.

### Statistical analyses

Continuous variables were described as mean (± standard deviation [SD]) or median (interquartile range [IQR]). Normal distribution was assessed with the Shapiro–Wilk test and normal Q-Q plot. Categorical variables were presented as percentages. Differences between groups were assessed using the Student’s T-test or Mann–Whitney U test, as appropriate, for continuous variables and the Chi-square test for categorical variables. Curves for mortality and hospital readmission were generated by the Kaplan–Meier method and compared with the log-rank test.

Stepwise Cox proportional hazards regression were performed to identify independent markers of in-hospital and 1-year mortality. The analyses included age, sex, the target variable (age-adjusted NT-proBNP levels) and all clinically relevant covariates with *p* < 0.2 in the univariate analysis. An AUROC analysis was performed to identify the best cut-off values of baseline NT-proBNP to predict mortality. A post-hoc sample size calculation was also performed for a two-side comparison, pre-specified alpha error of 0.05, pre-specified power of 80% (Supplementary material 1).

All statistical analyses were performed with R version 3.6.1 (R Foundation for Statistical Computing, Vienna, Austria). Statistical significance was set at *p* ≤ 0.05.

## Results

### Patients

**Fig. 1:**
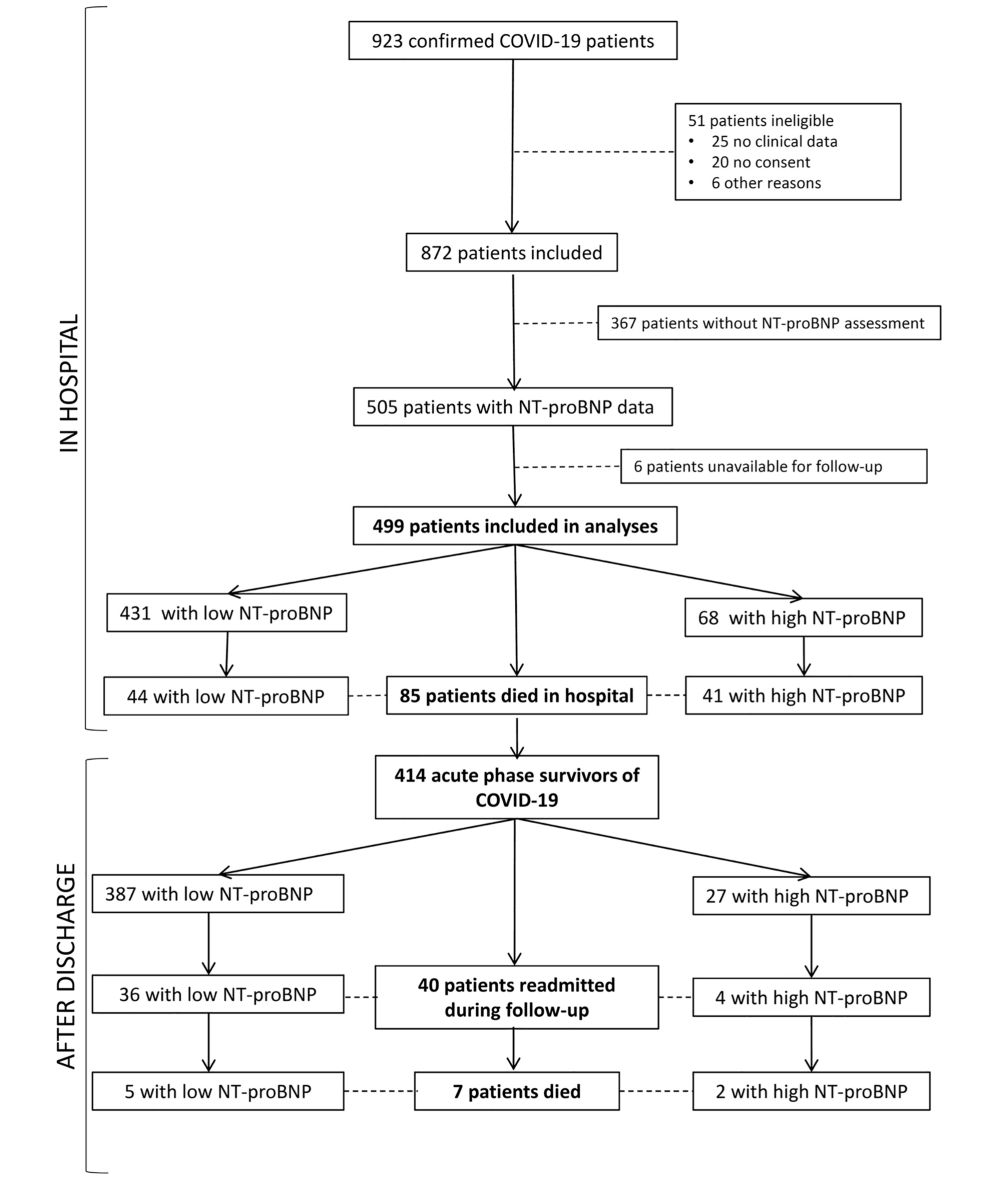
Flow chart showing patient inclusion in the study

From February to April 2020, a total of 923 patients were diagnosed with COVID-19 in our hospital. NT-proBNP was assessed at hospital admission in 505 (54.7%). Six of these patients were excluded from the study because they were not from our area and would not be available for follow-up. The remaining 499 patients were included and were followed up for 13.2 (range, 12.6–14.1) months after initial hospital admission; 414 of these patients survived the acute phase of COVID-19 (Fig. 1).

The baseline characteristics for all 499 patients are shown in Table 1. The median NT-proBNP value for all patients was 163 pg/mL. Sixty-eight patients had high age-adjusted NT-proBNP levels and 431 had low levels. Patients with high NT-proBNP levels were older, had a higher prevalence of cardiovascular risk factors such as hypertension and diabetes mellitus, and had more comorbidities, including CKD, coronary disease, atrial fibrillation, and COPD (Table 1). Basal treatment with ACEi/ARAII was also higher in the High NT-proBNP group (50% (34/68) vs. 31.3% (135/431, *p* = 0.002). They also had a higher prevalence of previous heart failure (20.6% vs. 2.8%; *p* < 0.001) and higher levels of inflammatory markers and myocardial injury, defined as hs-cTnT ≥14ng/L. In 53.3% of patients of the global cohort with prior treatment with ACEi/ARAII, the treatment was maintained during hospitalization, with no differences between high and low NTproBNP groups (*p* = 0.8).

**Table 1:** Baseline patient characteristics according to age-adjusted NT-proBNP levels

	All Patients n 499	High NT-proBNP n 68	Low NT-proBNP n 431	p*
	n (%)	n (%)	n (%)	
**Cardiovascular risk factors**				
Male sex	259 (52.0)	32 (47.1)	227 (52.7)	0.443
Age, yrs – median [IQR]	66 [52–77]	82.0 [74.0–88.0]	63.0 [50.5–75.0]	< 0.001
BMI, kg/m2 – median (IQR)	28.7 [25.6–32.8]	28.4 [25.5–33.5]	28.7 [25.7–32.6]	0.873
Ever smoked	107 (21.4)	17 (25.0)	90 (20.9)	0.707
Hypertension	245 (49.1)	57 (83.8)	188 (43.6)	< 0.001
Dyslipidemia	171 (34.3)	30 (44.1)	141 (32.7)	0.072
Diabetes mellitus	103 (20.7)	25 (36.8)	78 (18.1)	0.001
**Comorbidities**				
Cerebrovascular disease	32 (6.4)	10 (14.7)	22 (5.1)	0.006
Coronary heart disease	36 (7.2)	14 (20.6)	22 (5.1)	< 0.001
Atrial fibrillation	38 (7.6)	22 (32.4)	16 (3.71)	< 0.001
Chronic heart failure	26 (5.2)	14 (20.6)	12 (2.8)	< 0.001
Chronic kidney disease	48 (9.6)	26 (38.2)	22 (5.1)	< 0.001
Cancer	67 (13.4)	12 (17.6)	55 (12.8)	0.346
Peripheral vascular disease	15 (3.0)	7 (10.3)	8 (1.9)	0.002
COPD	39 (7.8)	12 (17.6)	27 (6.3)	< 0.003
**Laboratory blood tests – median** [**IQR)**				
Leukocytes, µL×10^3	6.5 [4.98–8.44]	8.55 [6.54–11.7]	6.30 [4.84–8.04]	< 0.001
Lymphocytes, µL×10^3	1.06 [0.74–1.50]	0.80 [0.49–1.18]	1.10 [0.79–1.54]	< 0.001
Creatinine, mg/dL	0.9 [0.71–1.12]	1.25 [0.98–1.80]	0.87 [0.68–1.06]	< 0.001
Haemoglobin, g/dL	13.6 [12.3–14.6]	12.4 [10.9–13.4]	13.8 [12.5–14.7]	< 0.001
LDH, U/L	287 [233–378]	392 [272–501]	281 [228–362]	< 0.001
C-reactive protein, mg/dL	7.85 [3.18–14.2]	11.2 [5.20–24.0]	7.10 [3.00–13.0]	< 0.001
D-Dimer, ng/mL	700 [440–1232]	1130 [680–2365]	670 [426–1100]	< 0.001
hs-cTnT, ng/L	0 [0-19.6]	50.7 [29.7–100]	0.00 [0.00-14.3]	< 0.001

IQR, interquartile range; BMI, body mass index; COPD, chronic obstructive pulmonary disease; LDH, lactate dehydrogenase; hs-cTnT, high sensitive cardiac-specific troponin T

*p-value for the comparison between patients with high vs. low NT-proBNP levels

### NT-proBNP and in-hospital complications and mortality

Patients with high NT-proBNP levels had a higher incidence of acute heart failure and a greater need for non-invasive and invasive mechanical ventilation (Table 2). They also showed a non-significant trend towards more cases of new-onset arrhythmia (6.35% vs. 2.39%; *p* = 0.09).

**Table 2:** In-hospital complications and mortality for all patients and according to age-adjusted NT-proBNP levels

	All Patients n 499	High NT-proBNP n 68	Low NT-proBNP n 431	
	n (%)	n (%)	n (%)	p*
Acute heart failure	25 (5.0)	17 (25)	8 (1.9)	< 0.001
Need for non-invasive mechanical ventilation^^^	32 (6.4)	10 (14.7)	22 (5.1)	< 0.001
Need for invasive mechanical ventilation	45 (9.0)	14 (20.6)	31 (7.2)	< 0.001
Mortality	85 (17.0)	41 (60.3)	44 (10.2)	< 0.001

^^^ Non-invasive mechanical ventilation included high-flow nasal cannula and non-invasive ventilation

*p-value for comparison between patients with high vs. low NT-proBNP levels

A total of 85 patients (17%) died in hospital: 41 (60.3%) with high NT-proBNP levels and 44 (10.2%) with low levels (*p* < 0.001) (Fig. 1). The multivariate analysis identified high age-adjusted NT-proBNP levels as an independent marker of in-hospital mortality (HR 1.95, 95% CI 1.07–3.53) (Table 3).

**Table 3:** Multivariate Cox regression analysis for in-hospital mortality

	HR	95% CI	*p*
High NT-proBNP	1.95	1.07–3.53	0.027
Age	1.09	1.06–1.12	< 0.001
hs-cTnT^+^	1.72	1.33–2.22	< 0.001
C-reactive protein	1.04	1.02–1.07	< 0.001
Chronic kidney disease	2.77	1.50–5.12	0.001

Adjusted by: sex, hypertension, diabetes, chronic heart failure, ischemic heart disease, atrial fibrillation or flutter, chronic kidney disease, COPD, respiratory support, D-dimer, C-reactive protein, and hs-cTnT

^+^TnT, per 100ng/L

Median hospital stay was ten days for all patients, with no significant differences between those with high vs. low NT-proBNP levels (*p* = 0.43).

In our serie, hydroxychloroquine was administered in 95% of the patients (96% in low NT-proBNP values group vs. 88.2% in high NT-proBNP values group, *p* = 0.012). Regarding corticosteroids, there were administered in 36.8% of the patients, with differences between the two groups (56.9% in high NT-proBNP values group vs. 33.7% in low NT-proBNP values, *p* = 0.001). Few antiretrovirals were used, following the available recommendations at that time, including Lopinavir/ritonavir (8.4% overall) or other treatments as Tocilizumab (15.6%) with no differences between groups.

### NT-proBNP and one-year mortality

**Fig. 2:**
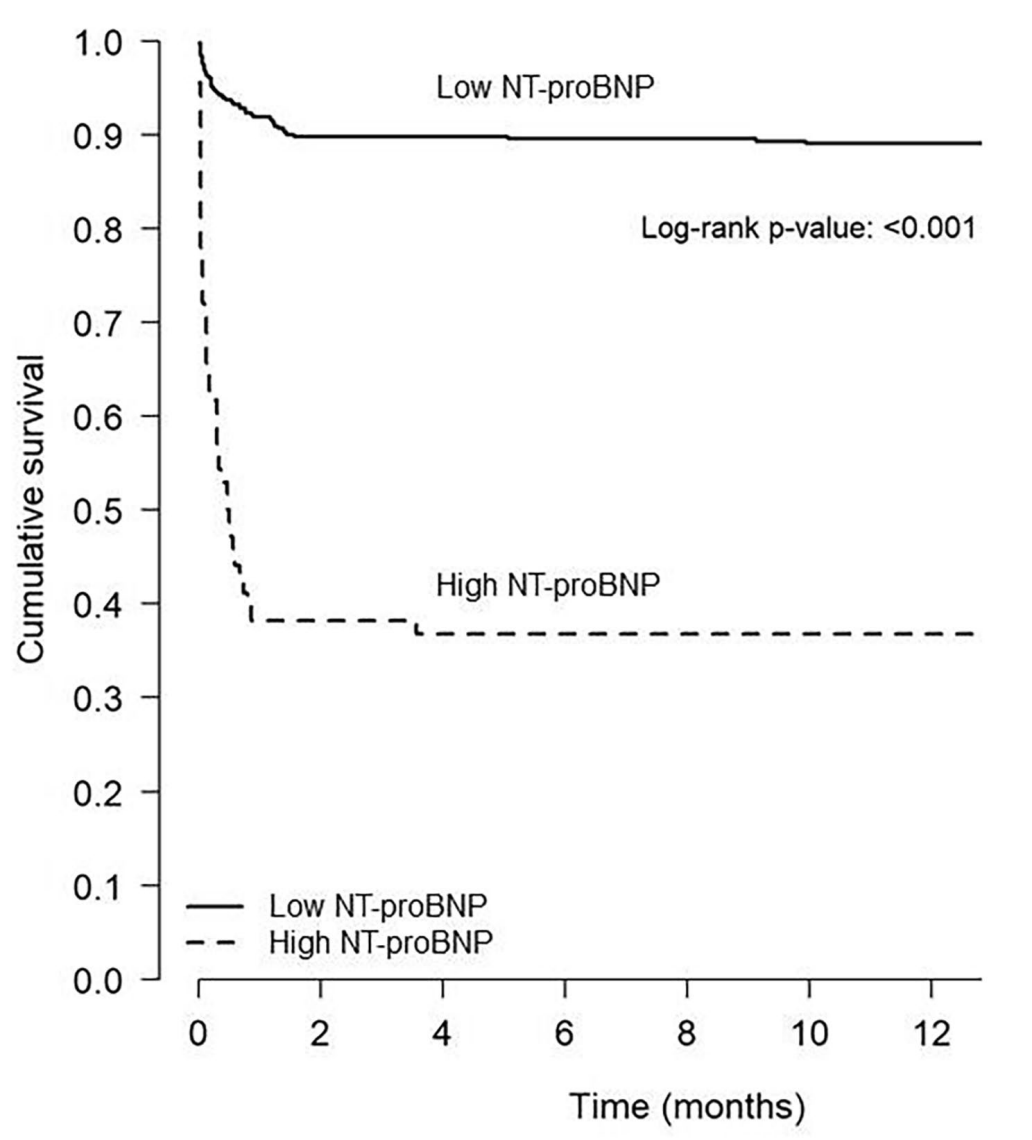
Kaplan Meier curves of mortality during the first year of COVID-19 infection according to age-adjusted NT-proBNP levels

Of the 499 patients included in the study, 92 died within the first year after initial hospital admission (85 during hospitalization and seven after discharge), with a significantly higher percentage of deaths among patients with high NT-proBNP levels than among those with low levels (63.2% vs. 11.4%; *p* < 0.001) (Figs. 1 and 2).

The mean NT-proBNP value at initial hospital admission was significantly higher in patients who died within the first year than in those alive at one year (1264 vs. 109 pg/mL; *p* < 0.001). In addition, patients who died within the first year were older and had more cardiovascular risk factors and comorbidities (Table 4). The best NT-proBNP cut-off to predict 1 year mortality identified by the AUROC analysis was 455ng/ml with a sensitivity of 72.2% and a specificity of 82% (AUC 0.8355). The ROC curve is shown in Supplementary Fig. 1.

**Table 4:** Patient characteristics at hospital admission for all patients and according to vital status at one-year follow-up

	All Patients (n 499)	Alive (n 407)	Dead (n 92)	
	n (%)	n (%)	n (%)	p*
**Cardiovascular risk factors**				
Male sex	259 (51.9)	216 (53.1)	43 (46.7)	0.253
Age, yrs – median (IQR)	66.0 [52.0–77.0]	61.5 [50.0–73.0]	82.0 [75.0–88.0]	< 0.001
BMI, kg/m2 – median (IQR)	28.7 [25.6–32.8]	28.7 [25.3–32.5]	28.7 [26.2–33.7]	0.562
Ever smoked	107 (21.4)	86 (21.1)	21 (22.8)	0.773
Hypertension	245 (49.1)	179 (43.9)	66 (71.7)	< 0.001
Dyslipidemia	171 (34.3)	127 (31.2)	44 (47.8)	0.004
Diabetes mellitus	103 (20.6)	70 (17.2)	33 (35.9)	< 0.001
**Comorbidities**				
Cerebrovascular disease	32 (6.4)	18 (4.4)	14 (15.2)	< 0.001
Coronary heart disease	36 (7.2)	20 (4.94)	16 (17.4) )	< 0.001
Atrial fibrillation	38 (7.6)	20 (4.9)	18 (19.5)	< 0.001
Chronic heart failure	26 (5.2)	12 (2.9)	14 (15.2)	< 0.001
Chronic kidney disease	48 (9.6)	24 (5.9)	24 (26.1)	< 0.001
Cancer	67 (13.4)	48 (11.8)	19 (20.7)	0.035
Peripheral vascular disease	15 (3.0)	10 (2.5)	5 (5.4)	0.171
COPD	39 (7.8)	27 (6.6)	12 (13.0)	0.065
**Laboratory blood tests – median** [**IQR]**				
Leukocytes, µL×10^3	6.50 [4.98–8.44]	6.36 [4.90–8.05]	8.00 [5.23–10.4]	0.001
Lymphocytes, µL×10^3	1.06 [0.74–1.50]	1.12 [0.81–1.55]	0.76 [0.50–1.20]	< 0.001
Creatinine, mg/dL	0.90 [0.71–1.12]	0.87 [0.68–1.06]	1.09 [0.86–1.60]	< 0.001
Haemoglobin, g/dL	13.6 [12.3–14.6]	13.8 [12.6–14.7]	12.8 [11.2–14.0]	< 0.001
LDH, U/L	287 [233–378]	281 [229–359]	380 [258–461]	< 0.001
C-reactive protein, mg/dL	7.8 [3.18–14.2]	6.70 [2.90–12.6]	11.7 [5.20–22.8]	< 0.001
D-Dimer, ng/mL	700 [440–1232]	640 [420–1065]	1020 [690–2480]	< 0.001
hs-cTnT, ng/L	0.00 [0.00-19.6]	0.00 [0.00–14.0]	31.0 [17.1–64.6]	< 0.001
NT-proBNP. pg/mL	164 [42.7–514]	109 [32.2–336]	1264 [367–4717]	< 0.001

IQR, interquartile range; BMI, body mass index; COPD, chronic obstructive pulmonary disease; LDH, lactate dehydrogenase; hs-cTnT, high sensitive cardiac-specific troponin T

*p-value for the comparison between alive vs. dead patients at one year

High age-adjusted NT-proBNP levels were associated with higher 1 year mortality (HR 5.46, 95% CI 3.37–8.84; *p* < 0.001). After a multivariate analysis, high NT-proBNP levels remained as a predictor for adjusted mortality at one year (HR 2.69, 95% CI 1.47–4.89; *p* = 0.001) (Table 5).

**Table 5:** Multivariate Cox regression analysis of 1-year mortality

	HR	95% CI	*p*
High NT-proBNP	2.69	1.47–4.89	0.001
Age	1.08	1.06–1.11	< 0.001
Need for non-invasive mechanical^^^ ventilation^^^	4.04	0.95–17.12	0.057
Need for invasive mechanical ventilation	8.19	1.84–36.46	0.006
C-reactive protein	1.02	1.00-1.05	0.034

^^^ Non-invasive mechanical ventilation included high-flow nasal cannula and non-invasive ventilation

Adjusted by sex, hypertension, ever smoked, diabetes, chronic heart failure, ischemic heart disease, atrial fibrillation or flutter, chronic kidney disease, COPD, cancer history, respiratory support, D-dimer, C-reactive protein and hs-cTnT.

### NT-proBNP in survivors of the acute phase of COVID-19

Of the total of 499 patients, 414 survived the acute phase of COVID-19 (27 with high and 387 with low NT-proBNP levels). Baseline characteristics of acute-phase survivors are shown in Supplementary Table 1. Acute phase survivors with high NT-proBNP showed a greater tendency to die or be readmitted during follow-up (18.5% vs. 8.8%, *p* = 0.095) (Table 6) but without significance after multivariate adjusting (HR 2.20 [0.82–5.90]).

**Table 6:** Death and hospital readmission due to any cause during 1-year follow-up among survivors of acute-phase COVID-19.

	All n 414	High NT-proBNP n 27	Low NT-proBNP n 387	
	n (%)	n (%)	n (%)	p*
Death or hospital readmission	40 (9.7)	5 (18.5)	34 (8.8)	0.095
Hospital readmission for any cause	40 (9.7)	4 (14.8)	36 (9.3)	0.350
Death	7 (1.7)	2 (7.4)	5 (1.3)	0.018

*p-value for comparison between patients with high vs. low NT-proBNP

After hospital discharge, 40 of these patients (9.66%) were readmitted, with no significant differences between patients with high vs. low levels of NT-proBNP (*p* = 0.350). Only one patient with high levels and two with low levels were readmitted due to cardiac cause.

Among the 414 patients who survived the acute phase of COVID-19, seven had died at one year – two (7.4%) with high NT-proBNP levels and five (1.3%) with low levels of NT-proBNP (*p* = 0.018) (Table 6). Death was due to cardiovascular disease in one patient with low NT-proBNP levels but in no patient with high levels.

## Discussion

In the present study, we have analyzed a large prospective cohort of Spanish patients admitted to hospital for COVID-19 and found that nearly 14% of patients had high age-adjusted NT-proBNP levels at the time of hospital admission. NT-proBNP levels were significantly associated with both short and long-term prognosis. At the time of hospital admission, high levels were associated with older age, the presence of cardiovascular risk factors, and a higher prevalence of cardiovascular diseases and other comorbidities. Patients with high NT-proBNP levels had significantly more in-hospital complications, an elevated NT-proBNP was independently associated with in-hospital mortality (HR 1.95, 95% CI 1.07–4.89). In addition, NT-proBNP levels were identified as a prognostic marker of mortality at one year (HR 2.69, 95% CI 1.47–4.89). Finally, among survivors of the acute phase of COVID-19, there was an association between high NT-proBNP levels and death after hospital discharge (*p* = 0.018) and a tendency towards more readmission rates in patients with high NT-proBNP levels.

### NT-proBNP assessment

Other studies, in different patient populations and using different cut-off points, have reported high NTproBNP levels in 15–50% of COVID-19 patients [17, 18, 24]. In the present study, we used age-adjusted cut-off points [21–23] to classify our patients and found that 13.6% had high NT-proBNP levels. Several studies have attempted to establish the best cut-off point of NT-proBNP to predict mortality in COVID-19 patients, but results have not been consistent [25]. Furthermore, levels can vary depending on when they are assessed – at hospital admission or during hospitalization [26–28]. In the present study, the median NT-proBNP level at admission for the entire cohort was 163pg/mL, while levels were clearly higher in patients who died (1264pg/mL).

The mechanisms underlying an increase of NT-proBNP are not entirely known. BNPs are primarily produced in the heart and released into the circulation in response to increased wall tension in both the atria and ventricles [29, 30]. However, elevated NT-proBNP can result not only from cardiac causes but also from impaired renal function, atrial fibrillation, older age, malnutrition (low albumin), or high levels of C-reactive-protein [29, 30]. Hypoxia and certain hormones, such as catecholamines, angiotensin II, and endothelin, can stimulate secretion of BNP [31]. In addition, ventricular wall stress can be increased by hypoxia-induced pulmonary hypertension and vasopressors, significant inflammation due to cytokine storm, and renal failure. Direct heart damage in sepsis or in COVID-19 infection is also common [12, 15], and the volume resuscitation recommended in this setting may contribute to acute heart failure with a subsequent increase in NT-proBNP levels.

### NT-proBNP and COVID-19: short-term prognosis

Previous studies of COVID-19 patients have found that elevated NT-proBNP is associated with worse outcomes during hospitalization, including higher mortality rates [17–20]. Along the same lines, 60.3% of our patients with high NTproBNP levels died during hospitalization, compared to 10.2% of patients with low levels. In addition, patients with high NT-proBNP had more complications during hospitalization, including acute heart failure and a need for mechanical ventilation. In previous studies about pneumonia and SARS in critically ill patients, high NT-proBNP levels were also found to be a good predictor of complications [11, 32].

Many previous studies have associated myocardial injury, defined as an elevation of cardiac troponin, with adjusted mortality of SARSCov2 patients (9, 14–16). In fact, high age-adjusted NT-proBNP levels at the time of admission were predictive of in-hospital mortality independently of hs-cTnT values. These results are in line with those of Caro-Codón et al., who found that high age-adjusted NT-proBNP was independently associated with death during the first two months after COVID-19 diagnosis [18]. Another study in Argentinian patients found that NT-proBNP > 300pg/mL was an independent predictor of mortality while troponin was not [27].

Taken together, these findings indicate that NT-proBNP is a useful tool to predict short-term prognosis in COVID-19 patients although the optimal cut-off point for NT-proBNP levels has yet to be established.

In our cohort, while differences were observed in the administration of hydroxychloroquine or corticosteroids among those with baseline high or low NT-proBNP values, subsequent studies have revealed that the use of hydroxychloroquine in the acute phase does not reduce mortality [33–34], nor do corticosteroids benefit non-severe patients [33, 35]. As a result, their widespread use is no longer recommended. Furthermore, since they were patients from the first wave, there were no other available drugs that could have modified survival in the acute phase. Despite initial uncertainty regarding whether the need to discontinue ACE inhibitors, angiotensin receptor blockers (ARBs) or Neprilysin inhibitor -Angiotensin II receptor blocker combination (ARNi), subsequent observations have suggested potential benefits [36, 37]. In our center, the treatment with ACE inhibitors/ARBs/ARNi was not discontinued according to the hospital protocol if patient’s clinical situation permitted it.

### NT-proBNP and COVID-19: long-term prognosis

In the present study, high NT-proBNP levels were associated with a higher one-year mortality rate (63.2% vs. 11.4%; *p* < 0.001) and emerged as an independent marker of one-year mortality in the multivariate analysis (HR 2.69, 95% CI 1.47–4.89; *p* = 0.001), although these differences were driven mainly by in-hospital deaths. Among survivors of COVID-19 hospitalisation, high NT-proBNP levels showed an association with high mortality rates (*p* = 0.018), though the low number of events in these patients (*N* = 7/414) makes it difficult to draw definite conclusions. Along these lines, Sabanoglu et al. also included in-hospital deaths and found that NT-proBNP ≥ 1008pg/mL was a good predictor of one-year mortality [38]. Llàcer et al. investigated the mid-term prognosis (median follow-up of 169 days) of COVID-19 patients and found that patients with previous heart failure constituted a special high-risk subgroup of patients, with more adverse events [39]. In our study, patients with high NT-proBNP levels had more previous history of heart failure and a higher incidence of heart failure during hospitalization.

High hs-cTnT levels in the acute phase of COVID-19 have been associated with worse long-term prognosis [14, 33] but there is limited data on the potential association between NT-proBNP levels in the acute phase and long-term prognosis. Our findings indicate that high NT-proBNP levels may be able to identify patients with a high risk of complications both during and after hospitalization for COVID-19 infection. Further investigation is warranted to validate our results, especially regarding long-term prognosis in acute-phase survivors, and to elucidate the mechanism of this association. It is worth nothing that the majority of the readmission in both groups were non cardiac.

Our study has several limitations. Firstly, it was performed in a single center, which may limit the generalizability of our findings to a broader population; however, our results are in line with those of other studies. Secondly, the absence of available NT-proBNP data for all patients treated in our center could introduce a potential bias. However, the final percentage of patients included is substantial taking into account the exceptional situation of the sanitary emergency and we consider it representative. The results align with findings of other research groups [38], nevertheless, further studies are necessary to confirm these results. Thirdly, the study was performed during the first wave of COVID-19 and we cannot exclude the possibility that vaccination, antiretroviral therapies or other beneficial treatments could affect the results currently.

Fourthly, the small number of events in our patients who survived the acute phase of COVID-19 may have limited the statistical power of our study and the conclusions that can be drawn. Lastly, it would have been informative to perform a second assessment of NT-proBNP during follow-up but this was impossible due to pandemic-related restrictions on hospital visits.

## Conclusions

High age-adjusted NT-proBNP levels at the time of hospital admission for COVID-19 are associated with a higher incidence of in-hospital complications and death and with poor short- and long-term prognosis. NT-proBNP may also be a good predictor of worse prognosis in survivors of the acute phase of COVID-19. Therefore, a close follow-up in patients with high NT-proBNP levels after their hospital discharge seems reasonable. Further research is warranted to confirm the impact of NT-proBNP on long-term prognosis in acute-phase survivors.

### Electronic supplementary material

Below is the link to the electronic supplementary material.

Supplementary Material 1

## Data Availability

The datasets used and analyzed during the current study are available from the corresponding author on reasonable request.

## Abbreviations

ARDS: Acute respiratory distress syndrome
ARBs: Angiotensin receptor blockers
ARNi: Neprilysin inhibitor -Angiotensin II receptor blocker combination
BMI: Body mass index
CI: Confidence interval
CKD: Chronic kidney disease
COPD: Chronic obstructive pulmonary disease
COVID-19: Coronavirus disease 2019
Hs-cTnT: High-sensitive troponin T
IQR: Interquartile range
NT-proBNP: N-terminal pro B-type natriuretic
SARS-CoV-2: Severe acute respiratory syndrome coronavirus 2
SD: Standard deviation
