# Supplementary material for: Baseline NT-proBNP levels as a predictor of short-and long-term prognosis in COVID-19 patients: a prospective observational study
Source: BMC Infect Dis. 2024 Jan 8;24:58. doi: 10.1186/s12879-024-08980-3 (PMC10773093; doi:10.1186/s12879-024-08980-3)
Supplement: Supplementary file 1 — Supplementary Material 1 [file 12879_2024_8980_MOESM1_ESM.docx]

**Supplementary Post-hoc sample size calculation**

We have conducted a post-hoc estimation of the sample size for the primary endpoint of our study, 1-year mortality:

For a two-side comparison, pre-specified alpha error of 0.05, pre-specified power of 80%, a prevalence of 13.6% of patients with high NT-proBNP and a HR of 2.69 according with our study, we would need to enroll 107 participants. Our actual sample size was larger than this estimate.

Moreover, we found interesting to estimate a post-hoc sample size for the secondary combined endpoint, hospital readmission or mortality after hospital discharge in patients who survived the acute phase of COVID-19:

For a two-side comparison, pre-specified alpha error of 0.05, pre-specified power of 80%, with a prevalence of 6.5 % of patients with high NT-proBNP and a pre-specified HR of 1.5 considered clinically relevant, we would need to enroll 924 participants.

**Supplementary Figure 1. ROC curve of NT-proBNP for 1-Year Mortality.**

**
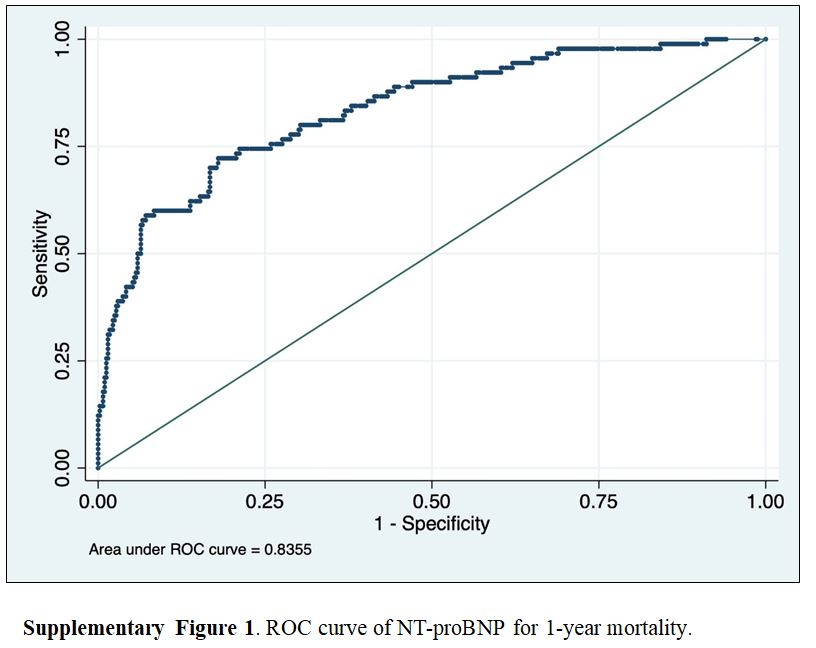
**

**Supplementary table 1.**

| **Table 1 SM.**  Baseline characteristics at hospital admission in survivors according to NT-proBNP status. | | | | |
| --- | --- | --- | --- | --- |
|  | **All Patients**  **n 414** | **High NT-proBNP**  **n 27** | **Low NT-proBNP**  **n 387** |  |
|  | **n (%)** | **n (%)** | **n (%)** | **p-value*** |
| ***Cardiovascular risk factors*** | | | | |
| Male sex, n (%) | 221 (53.4) | 16 (59.3) | 205 (52.9) | 0.537 |
| Age, years * | 62 [50-74] | 74.0 [65.5;81.0] | 61.0 [50.0;72.0] | 0.001 |
| Body mass index, kg/m2 [BMI] | 28.7 [25.6-32.5] | 28.7 [26.7;35.1] | 28.7 [25.4;32.4] | 0.493 |
| Ever smoked, n (%) | 91 (22.0) | 8 (29.6) | 83 (21.4) | 0.382 |
| Hypertension, n (%) | 183 (44.2) | 21 (77.8) | 162 (41.9) | <0.001 |
| Dyslipidemia, n (%) | 130 (31.4) | 11 (40.7) | 119 (30.7) | 0.312 |
| Diabetes mellitus, n (%) | 70 (17.0) | 9 (33.3) | 61 (15.8) | 0.026 |
| ***Comorbidities*** | | | | |
| Cerebrovascular disease, n (%) | 19 (4.6) | 3 (11.1) | 16 (4.1) | 0.109 |
| Coronary heart disease, n (%) | 22 (5.3) | 6 (22.2) | 16 (4.1) | 0.001 |
| Atrial fibrillation, n (%) | 22 (5.3) | 8 (29.6) | 14 (4.1) | <0.001 |
| Chronic heart failure, n (%) | 12 (2.9) | 2 (7.4) | 10 (2.6) | 0.172 |
| Chronic kidney disease, n (%) | 24 (5.8) | 7 (25.9) | 17 (4.4) | <0.001 |
| Cancer, n (%) | 52 (12.6) | 3 (11.1) | 49 (12.7) | 1.000 |
| Peripheral vascular disease, n (%) | 10 (2.4) | 3 (11.1) | 7 (1.8) | 0.020 |
| COPD, n (%) | 30 (7.2) | 5 (18.5) | 25 (6.5) | 0.032 |
| ***Laboratory blood tests*** | | | | |
| Leukocytes /µL×10^3* | 6.36 [4.9;8.11] | 8.28 [6.06;11.2] | 6.30 [4.84;8.00] | 0.002 |
| Lymphocytes /µL×10^3* | 1.12 [0.8;1.56] | 1.01 [0.83;1.34] | 1.12 [0.80;1.57] | 0.495 |
| Creatinine (mg/dL)* | 0.87 [0.68;1.06] | 1.10 [0.91;1.69] | 0.85 [0.68;1.05] | <0.001 |
| Haemoglobin (g/dL)* | 13.8 [12.6;14.7] | 12.8 [11.1;13.5] | 13.8 [12.7;14.7] | 0.001 |
| LDH (U/L)* | 280 [228;359] | 329 [246;440] | 279 [225;355] | 0.030 |
| C-reactive protein (mg/dL) * | 6.75 [2.9;12.7] | 7.75 [4.05;19.2] | 6.75 [2.90;12.2] | 0.155 |
| D-Dimer (ng/mL)* | 640 [420;1070] | 745 [512;1245] | 640 [410;1052] | 0.310 |
| hs- cTnT (ng/L) * | 0 [0;14.1] | 34.1 [19.9;62.4] | 0.00 [0.00;14.0] | <0.001 |
| *Values are expressed as median [IQR]. COPD, chronic obstructive pulmonary disease; LDH lactate dehydrogenase; hs-cTnT high-sensitivity cardiac-specific troponin T. | | | | |
